# The Impact of Vascular Access Types on Hemodialysis Patient Long-term Survival

**DOI:** 10.1038/s41598-019-47065-z

**Published:** 2019-07-24

**Authors:** Li-Mei Yeh, Sherry Yueh-Hsia Chiu, Ping-Chin Lai

**Affiliations:** 1 Hemodialysis unit, Department of Nephrology, Chang Gung Memorial Hospital, Taipei, Taiwan; 2 Department of Health Care Management and Healthy Aging Research Center, Chang Gung University, Taoyuan, Taiwan; 3 Division of Hepatogastroenterology, Department of Internal Medicine, Kaohsiung Chang Gung Memorial Hospital, Kaohsiung, Taiwan; 4 Department of Nephrology, Chang Gung Memorial Hospital, Taoyuan, Taiwan; 5 The Kidney Institute and Division of Nephrology, China Medical University Hospital, Taichung, Taiwan

**Keywords:** Prognosis, Kidney

## Abstract

Vascular access (VA) is the cornerstone for carrying out hemodialysis, yet it may bring in complications and leads to hemodialysis quality decline. This study aimed to explore the impact of vascular access types, including arteriovenous shunts and central venous catheter on all-cause mortality after adjustment of other risk factors. Total 738 ESRD patients aged over 40 year old receiving regular hemodialysis therapies were recruited between January 2001 and December 2010 from a single hemodialysis center in northern Taiwan. We ascertained the causes and date of death by linking our hospital database with Nationwide Mortality Registry Database. VA types and biochemistry parameters were extracted from the electronic hospital records. Patients were categorized into three groups, including (1)arteriovenous shunts (AVF)/arteriovenous shunts with Gortex®(AVG); (2)AVF/AVG combined central venous catheter; (3)catheter only. The time-dependent influence of vascular types i.e. initiation and follow-up period was also assessed. The mean follow-up time was 4.5 years. In patients using central venous catheter for initiation of hemodialysis, the adjusted hazard ratio (HR) for all-cause mortality was 1.55(95%CI: 1.09, 2.21), when compared with AVF/AVG. In the follow-up period, after adjustment for other risk factors, the multivariable analysis showed that the adjusted HRs were 3.23(95%CI: 1.85, 5.64) and 1.45(95%CI: 1.11, 1.91) for catheter only and AVF/AVG plus catheter, respectively. Our results showed that vascular accesses used for hemodialysis had different and time-dependent impact on patients’ long-term survival. Patients who started hemodialysis with central venous catheter had significantly higher all-cause mortality rate. Furthermore, in the follow-up period, patients both in the catheter only and AVF/AVG plus catheter groups also had the significant all-cause mortality rates. Our results support the early establishment of arteriovenous shunt for the chronic kidney disease patients.

## Introduction

The incidence of chronic renal failure is still rising against the continuing improvement of life expectancy. Once entering into stage 4–5 chronic kidney disease, patients need renal replacement therapy to maintain their daily activities. Hemodialysis is one of the treatment modalities these patients could choose among the three commonly used therapies including peritoneal dialysis and kidney transplantation. In some area, it is the most popular option. However, the overall mortality rate of hemodialysis patients is still high^1^ and several studies had been done to explore the underlying causes^2–5^. While diabetes mellitus, malnutrition and inflammation have been identified as the risk factors, some researchers found that vascular access also had an effect on the mortality of hemodialysis patients^6,7^. Still, the long term effect of vascular access on hemodialysis patient has not been investigated. Therefore, in this study, by using data from a single medical center in northern Taiwan, we aimed to explore the long term effect of vascular access on hemodialysis patient mortality.

## Results

Total 738 patients, aged 40–79 years, were recruited into this study. The follow-up period ranged from 0.3 to 10.9 years, with a mean of 4.5 years. Among these patients, 243 deaths were confirmed. The mortality rates by baseline vascular access types were 59.6, 96.4, and 181.6 per 1,000 person-year for AVF/AVG only, AVF/AVG plus catheter and catheter only, respectively. The catheter only patients had significantly higher mortality rate than the rest two groups of patients (AVF/AVG only and AVF/AVG plus catheter). More importantly, the AVF/AVG plus catheter patients also had significantly higher mortality rates than AVF/AVG only patients. Besides the vascular access types, the overall mortality rates were significantly higher among elder, high high-sensitivity CRP (hsCRP), diabetes mellitus, lower albumin, and high AST/ALT patients. The detail results were demonstrated in Table 1.

**Table 1:** The distribution of subjects’ baseline data and characters.

Variable	Classification	Subject	Death	Death%	Person-year	All-cause Mortality rate (per 1000)
**Initial** vascular Access type	AVF/AVG	599	202	33.7%	2879.36	70.15 (60.48,79.82)
	Catheter	139	41	29.5%	402.34	101.90 (70.71,133.09)
Vascular Access type	AVF/AVG only	436	130	29.8%	2182.43	59.6 (49.3,69.8)
	AVF/AVG plus catheter	270	98	36.3%	1016.66	96.4 (77.3, 115.5)
	Catheter only	32	15	46.9%	82.61	181.6 (89.7, 273.5.8)
Age group	40–49	111	12	10.8%	610.81	19.7 (8.5, 30.8)
	50–59	260	67	25.8%	1203.79	55.7 (42.3, 69.0)
	60–69	191	70	36.7%	862.95	81.1 (62.1, 100.1)
	70–79	176	94	53.4%	604.13	155.6 (124.1, 187.0)
Gender	Female	368	124	33.7%	1652.4	75.0 (61.8, 88.3)
	Male	370	119	32.2%	1629.3	73.0 (59.9, 86.2)
Fasting Plasma Glucose(mg/dl)	<126	415	109	26.3%	1962.4	55.5 (45.1, 66.0)
	$\ge$126	323	134	41.5%	1319.3	101.6 (84.4, 118.8)
Albumin (g/dl)	$\ge$3.5	537	161	30.0%	2516.7	64.0 (54.1, 73.9)
	<3.5	201	82	40.8%	765.0	107.2 (84.0, 130.4)
hsCRP (mg/L)	<4	426	116	27.2%	2062.7	56.2 (46.0, 66.5)
	$\ge$4	281	110	39.2%	1132.9	97.1 (79.0, 115.2)
	NK	31	17		86.0	197.6 (103.7, 291.5)
Cholesterol (mg/dl)	<240	703	228	32.4%	3108.2	73.4 (63.8, 82.9)
	$\ge$240	35	15	42.9%	173.5	86.5 (42.7, 130.2)
Triglyceride (mg/dl)	<200	544	158	29.0%	2384.2	66.3 (55.9, 76.6)
	$\ge$200	194	85	43.8%	897.5	94.7 (74.6, 114.8)
Uric acid (mg/dl)	<8	510	155	30.4%	2194.2	70.6 (59.5, 81.8)
	$\ge$8	226	87	38.5%	1082.1	80.4 (63.5, 97.3)
	NK	2	1			
AST (IU/L)	<40	698	222	31.8%	3150.4	70.5 (61.2, 79.7)
	$\ge$40	40	21	52.5%	131.3	159.9 (91.5, 228.3)
ALT (IU/L)	<40	693	224	32.3%	3105.1	72.1 (62.7, 81.6)
	$\ge$40	45	19	42.2%	176.6	107.6 (59.2, 156.0)
URR (%)	$\ge$65%	607	197	32.5%	2723.1	72.4 (62.3, 82.5)
	<65%	126	43	34.1%	535.00	80.4 (56.4, 104.4)
	NK	5	3			
Hb (g/dL)	$\ge$8.5	566	186	32.9%	2418.1	76.9 (65.9, 88.0)
	<8.5	172	57	33.1%	863.7	66.0 (48.9, 83.1)
Hct (%)	$\ge$28	437	144	33.0%	1801.3	79.9 (66.9, 93.0)
	<28	301	99	32.9%	1480.4	66.9 (53.7,80.0)

AVF: arteriovenous fistula; AVG: arteriovenous graft; URR: Urea reduction ratio; AST: aspartate aminotransferase; ALT: alanine aminotransferase; hs-CRP: high-sensitivity C-reaction protein; Hb: hemoglobin; Hct: hematocrit.

NK: not known.

**Figure 1:**
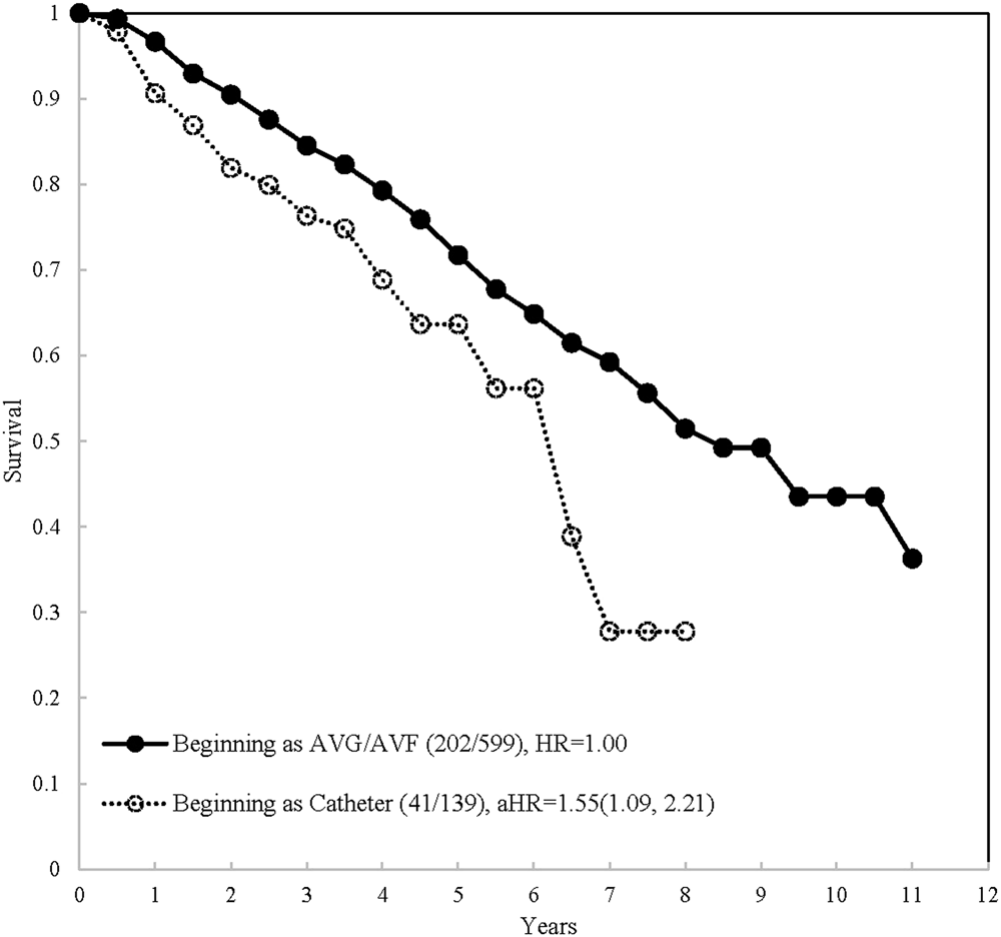
The survival of incident hemodialysis patients by initial vascular access types.

**Figure 2:**
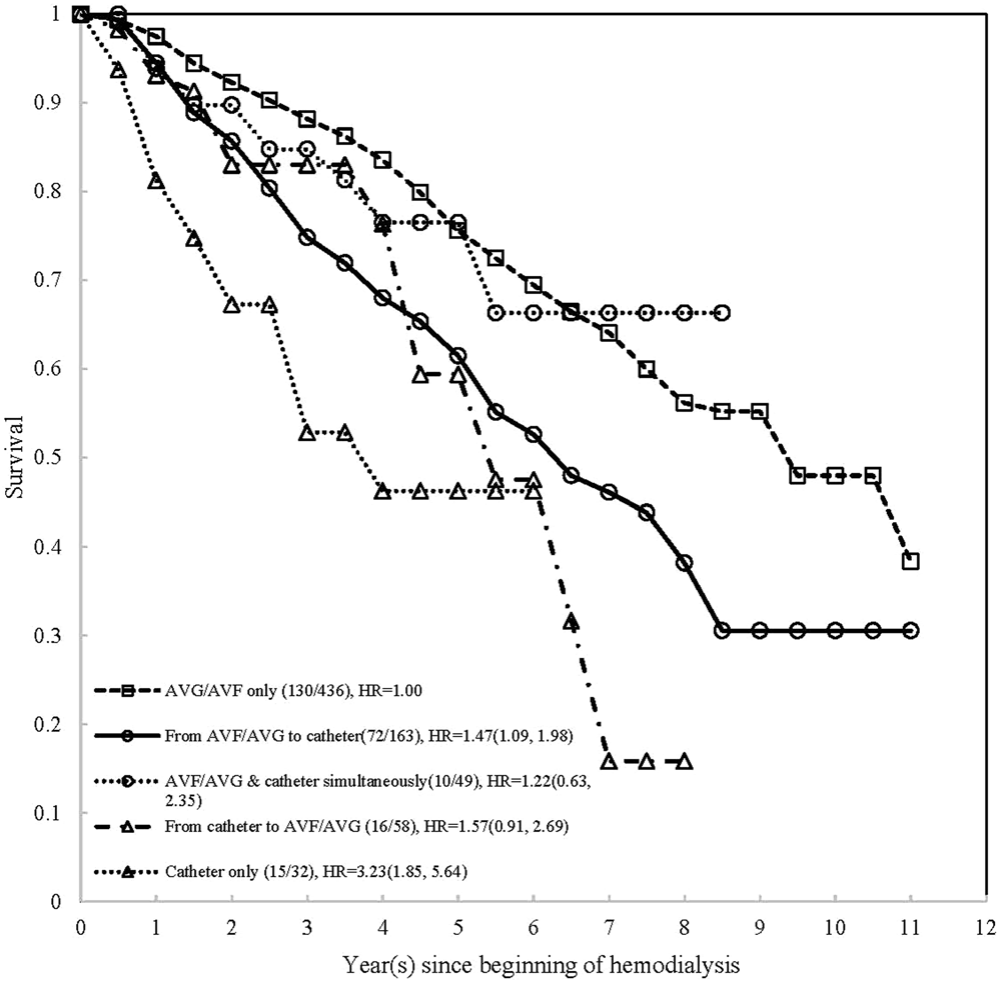
The survival of incident hemodialysis patients by 5 types of vascular access in the follow-up period.

**Figure 3:**
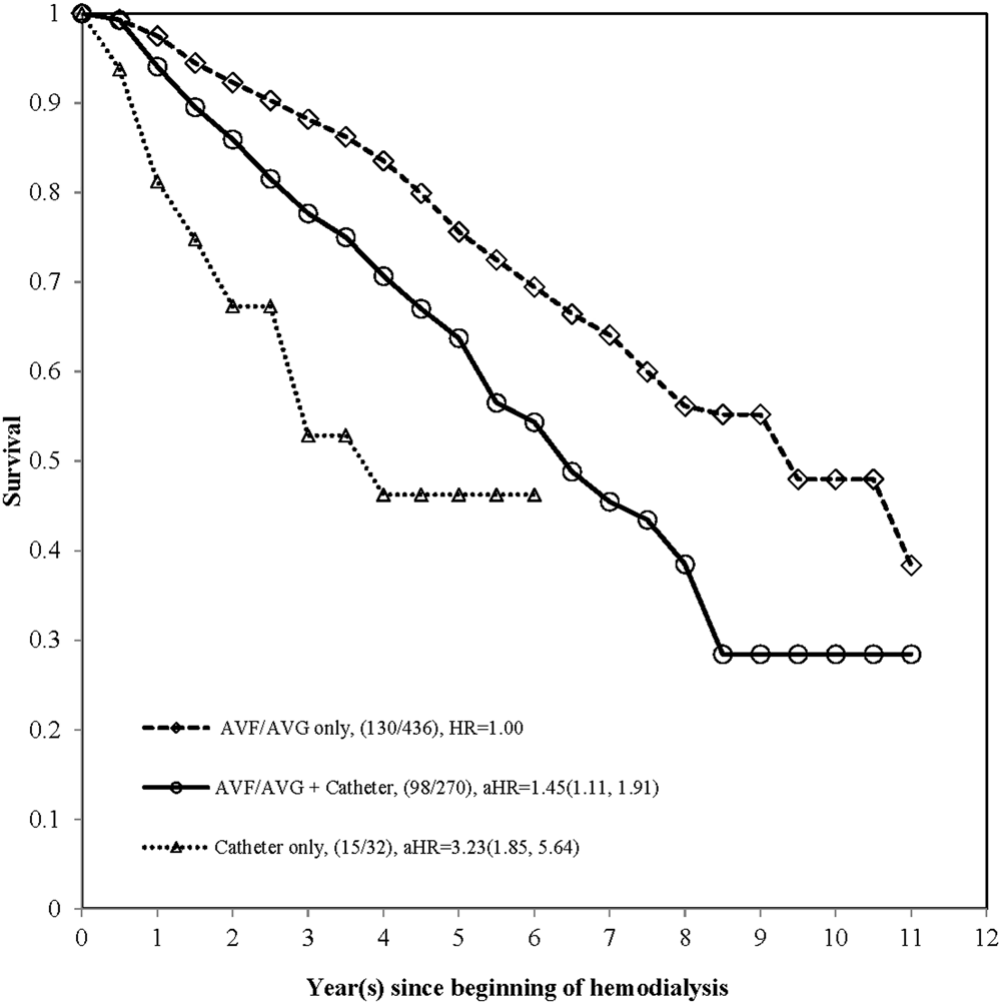
The survival of incident hemodialysis patients by vascular access types in the follow-up period.

Using Kaplan-Meier method, first, the survival curves of all-cause mortality were stratified by initial vascular access types and time points, which was demonstrated on Fig. 1. According to the exact time points of the use of AVF/AVG/catheter, a total of 436, 163, 49, 58, and 32 hemodialysis patients were classified into 5-type of vascular access as AVF/AVG only, from AVF/AVG to catheter, AVF/AVG & catheter simultaneously, from catheter to AVF/AVG, and catheter only, respectively (Fig. 2). The survival of AVF/AVG & catheter simultaneously and those who were from catheter to AVF/AVG were very close but with small sample size, therefore, both were combined into AVF/AVG→ catheter and defined as AVF/AVG plus catheter (Fig. 3). Based on our data, the checking by log-negative-log and Schoenfeld residuals was not violated the proportional hazards assumption (Supplement Figs S1 and S2). The all-cause mortality increased dramatically within 5-year among hemodialysis patients of AVF/AVG plus catheter and catheter only. The simultaneous multiple comparisons using Šidák correction adjustment also showed significant difference (p < 0.0001).

As shown above, we investigated the time dependent effect of vascular types on hemodialysis patient survival. The result showed that patients starting their hemodialysis with catheter had significantly higher HR for all-cause mortality when compared with patients starting with AVF/AVG (HR: 1.73 (95%CI: 1.23, 2.45)) (Fig. 1). Furthermore, regarding the vascular access change during follow-up, compared with AVF/AVG only, the crude HR were 1.84(95%CI: 1.38, 2.46), 1.26(95%CI: 0.66, 2.40), 2.11(95%CI: 1.24, 3.58), and 3.79(95%CI: 2.20, 6.52) for AVF/AVG→catheter, AVF/AVG & catheter simultaneously, catheter→AVF/AVG, and catheter only (Table 2). In the follow-up period with combined classification, we divided patients into three groups and found that the crude HRs of catheter only and AVG/AVF plus catheter were 3.79 (95%CI: 2.20, 6.52) and 1.80 (95%CI: 1.38, 2.34), respectively, when compared with AVF/AVG only (Table 3). By using univariate Cox proportional hazards regression, we found that significant risk factors for all-cause mortality among these subjects were age, diabetes (FPG≥126), lower albumin (<3.5), higher hsCRP (≥4), higher triglyceride (≥200), and higher AST (≥40), with HR 1.06(95%CI: 1.05, 1.08), 1.93(95%CI: 1.50, 2.49), 1.77(95%CI: 1.36, 2.32), 1.42(95%CI:1.09, 1.85), and 2.49(95%CI:1.59, 3.90) respectively (Table 3).

**Table 2:** The univariate and multivariable models of all-cause mortality risk of 5 types of vascular access on hemodialysis patients.

Variable	Classification	Univariate analysis		Multivariable analysis^#^	
		HR (95% CI)	p-valve	HR (95% CI)	p-valve
Vascular Access Type	AVF/AVG only	Reference	<0.0001	Reference	0.0004
	From AVF/AVG to catheter	1.84 (1.38, 2.46)		1.47 (1.09, 1.98)	
	AVF/AVG & catheter simultaneously	1.26 (0.66, 2.40)		1.22 (0.63, 2.35)	
	From catheter to AVF/AVG	2.11 (1.24, 3.58)		1.57 (0.91, 2.69)	
	Catheter only	3.79 (2.20, 6.52)		3.23 (1.85, 5.64)	

^#^Multivariable analysis adjusted age, gender, FPG, Albumin, hsCRP, AST.

**Table 3:** The univariate and multivariable models of all-cause mortality risk of different vascular access type on hemodialysis patients.

Variable	Classification	Univariate analysis		Multivariable analysis (1)		Multivariable analysis (2)	
		HR (95% CI)	p-valve	HR (95% CI)	p-valve	HR (95% CI)	p-valve
Initial vascular access	Catheter vs. AVF/AVG	1.73 (1.23, 2.45)	0.0018	1.55 (1.09, 2.21)	0.0150	—	—
Vascular Access type	AVF/AVG + catheter vs. AVF/AVG only	1.80 (1.38, 2.34)	<0.0001	—	—	1.45 (1.11, 1.91)	<0.0001
	Catheter only vs. AVF/AVG only	3.79 (2.20, 6.52)		—	—	3.23 (1.85, 5.64)	
Age		1.06 (1.05, 1.08)	<0.0001	1.06 (1.05, 1.08)	<0.0001	1.06 (1.05, 1.07)	<0.0001
Gender	Male/Female	0.98 (0.76, 1.26)	0.8555	1.24 (0.96, 1.60)	0.1024	1.28 (0.99, 1.66)	0.0588
FPG (mg/dl)	$\ge$126/<126	1.93 (1.50, 2.49)	<0.0001	1.77 (1.37, 2.29)	<0.0001	1.66 (1.28, 2.16)	0.0001
Albumin (g/dl)	<3.5/$\ge$3.5	1.77 (1.36, 2.32)	<0.0001	1.40 (1.06, 1.85)	0.0166	1.42 (1.08, 1.88)	0.0123
hsCRP (mg/L)	$\ge$4/<4	1.84 (1.41, 2.39)	<0.0001	1.48 (1.13, 1.93)	0.0041	1.43 (1.09, 1.87)	0.0094
Cholesterol (mg/dl)	$\ge$240/<240	1.17 (0.69, 1.97)	0.5609	—		—	
Triglyceride (mg/dl)	$\ge$200/<200	1.42 (1.09, 1.85)	0.0096	—		—	
Uric acid (mg/dl)	$\ge$8/<8	1.12 (0.93, 1.34)	0.2291	—		—	
AST (IU/L)	$\ge$40/<40	2.49 (1.59, 3.90)	<0.0001	2.69 (1.71, 4.23)	<0.0001	2.66 (1.68, 4.19)	<0.0001
ALT (IU/L)	$\ge$40/<40	1.52 (0.95, 2.44)	0.0790	—		—	
URR (%)	<65%/$\ge$65%	1.08 (0.96, 1.21)	0.2178	—		—	
Hb (g/dL)	<8.5/$\ge$8.5	0.82 (0.61, 1.10)	0.1824	—		—	
Hct (%)	<28/$\ge$28	0.78 (0.61, 1.01)	0.0632	—		—	

AVF: arteriovenous fistula; AVG: arteriovenous graft; URR: Urea reduction ratio; FPG: fasting plasma glucose; AST: aspartate aminotransferase; ALT: alanine aminotransferase; hsCRP: high-sensitivity C-reaction protein; Hb: hemoglobin; Hct: hematocrit.

NK: not known

Multivariable analysis (1): model for the initial use of vascular access type.

Multivariable analysis (2): model for the vascular access use in observed life-time.

In the multivariable Cox regression model, after adjustment for potential risk factors, our study revealed the adjusted HR of using catheter as the initial vascular access had significantly higher HR (1.55(95%CI: 1.09, 2.21)), when compared with AVG/AVF. In the light of vascular access change during follow-up, compared with AVF/AVG only, the adjusted HR were 1.47(95%CI: 1.09, 1.98), 1.22(95%CI: 0.63, 2.35), 1.57(95%CI: 0.91, 2.69), and 3.23(95%CI: 1.85, 5.64) for AVF/AVG→catheter, AVF/AVG & catheter simultaneously, catheter→AVF/AVG, and catheter only (Table 2). In the follow-up period, significantly higher HRs still existed among different subgroups of patients and the HRs were 3.23(95%CI: 1.85, 5.64) and 1.45(95%CI: 1.11, 1.91) for catheter only and AVF/AVG plus catheter respectively, when compared with AVF/AVG only. In multivariable model analysis, other risk factors were also identified including age, diabetes (FPG≥126), lower albumin (<3.5), higher hsCRP (≥4), and higher AST (≥40) with aHR1.06(95%CI: 1.05, 1.08), 1.77(95%CI: 1.37, 2.29), 1.40(95%CI:1.06, 1.85), 1.48(95%CI:1.13, 1.93), and 2.69(95%CI:1.71, 4.23), respectively (Table 3).

## Discussion

The prognosis of hemodialysis has made a vast improvement when compared with 1980s^1^. These progresses were mostly attributed to the introduction of erythropoietin^8^, calcitriol^9,10^ and new dialysis techniques^11^. However, the life quality and overall survival of hemodialysis patient are still inferior to those of kidney transplantation^12^. It had been reported that the survival of hemodialysis patient is seven times higher than general population^1^. To rectify this unfavorable situation, we need to explore what are the risk factors that contribute to the mortality of hemodialysis patients.

The URR (urea reduction ratio) indeed is one of the most widely recognized markers for hemodialysis adequacy and quality. In 1996, McClellan wet al. reported the 10% decrease on URR increased the mortality risk by 1.17-times^13^. However, in this study, we found that URR was not among the risk factors for mortality. We speculated that this might be due to similar URRs among these patients. To be exactly, the mean URR were 72.2%, 72.0%, and 76.2% for AVF/AVG only, AVF/AVG +catheter, and catheter only, respectively and the proportion of URR < 65% were 18%, 16%, and 10%. The other explanation is due to different confounding factors. Recently researches pointed out that elevation of inflammation biomarkers, such as C-reactive protein (CRP) was significantly associated with mortality^14^. However, in the McClellan report, those information were not included to adjust the morality risk. In this study, hsCRP level was included in the multivariable analysis to adjust the mortality risk.

Hyperlipidemia is a risk factor for atherosclerosis and coronary artery disease. It had been well documented that controlling serum cholesterol level significantly reduced the incidence of coronary artery diseases in general population. However in 4S study, treating hypercholesterolemia with statin in hemodialysis patients failed to demonstrate cardiovascular beneficial effects and improved all-cause mortality^15^. This might relate to multiple organs/pathways disorders occur in the hemodialysis patients. Therefore, hypercholestrolemia alone was not the risk factor for mortality. After adjustment for other potential risk factors, our study showed that patient with AST > = 40IU/L has higher risk of all-cause mortality when compared with patients had AST < 40 IU/L. It is worth to mention that ravel *et al*. in their 5-year follow-up study found similar results and demonstrated that patients with higher AST level had higher mortality rate. Serum AST level, not only represents the hepatitis status, but also reflects the abnormalities or damages on hemopoietic, cardiac, or muscle tissues.

Besides vascular access types, ours study also identified other risk factors such as age, diabetes, inflammation and hypoalbuminemia. Age had been mentioned in publication from other researcher as a risk factor for patient survival with older patients having worse survival^16^. Diabetes mellitus is a well-known risk factor for the survival of hemodialysis patients^2^. Diabetes per se could lead to the occurrence of macro- and micro-vasculopathy, which then result in cardiovascular and coronary artery diseases. Besides, both B and T cell functions could be compromised due to disturbance in glucose metabolism^17^. Therefore, the incidence of systemic infection and major adverse cardiac events increased in these patients, so is the mortality rate. Inflammation could occur in both sterile and non-sterile condition^18^. The release of pro-inflammatory cytokines such as TNF-alpha^18^, IL-6^19^ and IL-1 beta^20,21^ can increase catabolism, which then gives rise to energy-protein deficiency. A long term protein energy deficiency would manifest itself in malnutrition. This undernourishment in essential nutrients could elicit another spiral of infection and atherosclerosis and lead to further worsening in the general condition of these hemodialysis patients^4,5,22^.

To deliver adequate hemodialysis treatment, a vascular access with blood flow up-to 600 ml/min is the essence. Several types of vascular accesses are used in the clinical practice including, native arteriovenous fistula, synthetic polytetrafluoroethylene (Gortex®) arteriovenous fistula and central venous catheter with dual lumen. It has been shown that the native arteriovenous shunt could have fewer complications including infection episodes and longer durability than shunt built with synthetic material^23–25^. This advantage is even more prominent when compared with central venous catheter^24^. A fast and efficient arteriovenous shunt could offer a better clearance, while an unstable vascular access means under therapeutic effect. Thus, it is no surprise that the vascular access is one of the factors which determine short term prognosis of hemodialysis patients. However, fewer studies investigated the impact of vascular access on the long term survival of hemodialysis patients.

By using data from a single medical center, our study aimed to investigate the long term survival of hemodialysis patients who employed different vascular accesses to receive their regular hemodialysis. As shown in the result section, we found that patients who used central venous catheter as his vascular access throughout their courses had highest mortality rate, while patients who used arteriovenous shunts and never used any catheter had the lowest 10 years mortality rate. What surprised us is the result of patient using both catheter and arteriovenous shunt, the mortality rate of this group is significantly higher than the group used arteriovenous shunt only no matter the catheter is used initially or in the follow up period. These results strongly supported the idea that arteriovenous shunt should be established earlier in the chronic kidney disease patients to avoid using catheter as vascular access at any time. It is worth to mention that our results did not find URR and hyperlipidemia as risk factors for patient mortality. We think this is because most of the patients have URR greater than 65% and, if indicated, received statin treatment. Therefore, URR and hyperlipidemia were not different between survival and deceased patients.

Comparing to other studies, ours had the advantage of large patient population with reliable and detail records. These features made the results of this study closer to the daily clinical practice. The limit of this study including, first, this is a retrospective study; second, this is not a randomized study and biases may exist as patients with worst general condition tend to receive hemodialysis via central venous catheter; third, cardiac survey including cardiac echogram and blood pressure monitoring and residual renal function are not included in this study; fourth, these data all came from a tertiary referral medical center suggesting that these patients may have more systemic co-morbidities than patients in the primary hemodialysis facilities.

In conclusion, our study demonstrated that types of vascular access affected the long term survival of hemodialysis patients. Other risk factors such as diabetes, hypoalbuminemia and high hsCRP were also identified. Our results supported the early establishment of arteriovenous shunt in chronic kidney disease patients.

## Methods

### Study design and population

This retrospective cohort study was implemented to investigate the effect of vascular access (VA) on mortality. To be included in this study, the hemodialysis patients had to fulfill the following inclusion criteria: 1, aged 40–79 year-old; 2, receiving regular hemodialysis for more than three months in Chang Gung Memorial Hospital, LinKuo, Taiwan; 3, receiving hemodialysis three times a week and 4 hours per session. Between Jan 2001 and Dec 2010, a total 996 incident hemodialysis patients was ascertained from Hospital-based hemodialysis registry system, which recorded every detail of hemodialysis related data including the first and each subsequent dates of hemodialysis, biochemical parameters, and procedure types of vascular access, etc. Total 258 patients were excluded due to incomplete information of vascular access types and/or not receiving hemodialysis three times a week, 4 hours per session. The 738 patients fulfilling our inclusion criteria were analyzed. The study design and protocol were reviewed by Medical Ethics Committee and approved by Institute Review Board of Chang Gung Memorial Hospital, LinKuo, Taiwan (No. 99-0283B, 100–3983 C).

### Data collection

#### Types of vascular access

At current practice, three types of vascular accesses are used clinically. Hemodialysis patients’ vascular access can be formed either by their own vessel or artificial material. They are defined as AVF (Arteriovenous fistula) and AVG (Arteriovenous graft), respectively. The other is central venous catheter (CVC), which is established by inserting catheter into central vein with the other end fixed on the cutaneous tissue. In our center, our policy is to establish permanent arteriovenous shunt as early as possible in CKD stage 5 patients. Under emergency conditions such as hyperkalemia (serum potassium >6 meq/dL with clinical symptoms) or severe fluid overload, we would arrange emergent hemodialysis via catheter first and then performed arteriovenous shunt creation when clinical conditions allowed us to do that. Hemodialysis via catheter would never be our first choice if we can avoid it. Based on our hospital management system and nationwide health insurance claim, the exact dates of type use for hemodialysis were recorded for each procedure. Therefore, based on the sequential vascular access use by date, there were 5-type of vascular access classified into AVF/AVG only, from AVF/AVG to catheter, AVF/AVG & catheter simultaneously, from catheter to AVF/AVG, and catheter only.

These vascular accesses were recorded in both medical charts and hospital electronic database with date, surgery, and ICD code for each and every procedure. In this study, patients who received hemodialysis via central venous catheter throughout their dialysis courses in the follow-up period were categorized into catheter only group, while patients who used only AVF/AVG as their vascular access were classified into AVF/AVG group. Patient who used both AVF/AVG and central vein catheter for hemodialysis were put into AVF/AVG plus catheter group. The influence of vascular accesses on the patient survival was analyzed in two different time periods i.e. using catheter as the vascular access for the initiation of hemodialysis and in the follow-up period.

#### Baseline biochemical markers and definition

The patients’ baseline characteristics were collected from their first visit for hemodialysis and biochemical parameters were extracted from their routine quarter tests, which were performed by an automatic and standardized College of American Pathologists (CAP) approved central laboratory and recorded by hospital electronic system^26^. The prevalence of anti-HCV positivity was significantly higher in late stage of CKD/dialysis patients when compared with general population^27–29^. Ravel *et al*.^30^ found that higher AST level was associated with higher mortality in the maintenance hemodialysis patients with 5-year follow-up. We, therefore, included liver function tests, including AST and ALT, in our study. The definition for abnormal level of parameters were: fasting plasma glucose $\ge$126 mg/dl, albumin <3.5 g/dl, high-sensitivity C-Reactive Protein (hs-CRP) $\ge$4 mg/L, cholesterol $\ge$240 mg/dl, triglyceride $\ge$200 mg/dl, uric acid $\ge$8, AST  $\ge$ 40, ALT $\ge$ 40, hemoglobin (Hb) < 8.5 g/dl, Hct < 28%, and urea reduction ratio (URR) < 65%. The URR were calculated by (pre-dialysis urea level - post-dialysis urea level)/ pre-dialysis urea level that multiplied by 100%.

#### Causes and date of death and follow-up

The causes and date of death were ascertained from Taiwanese National Mortality Registry System which was a centralized database and maintained by Statistics Office, Ministry of Health and Welfare, Taiwan. All subjects in this cohort were followed-up till December 31, 2011. The cause and date of death of each patient’s was identified by linking hospital database with Nationwide Mortality Registry Database via each patient’s unique national identity number.

### Statistical analysis

The follow-up time in person-year was calculated from the first date of hemodialysis, which was systematic collected by hospital registry, to death or alive. For those still alive subjects, the last date of follow-up was December 31, 2011. All causes and date of death were retrieved from National Mortality Registry by Department of Statistics, Ministry of Health and Welfare, Taiwan. The Kaplan-Meier curves of all-cause mortality were stratified by different VA types and checked the simultaneous multiple comparisons with the Šidák correction adjustment^31^ (see Figs 1, 2 and 3).

Both log-negative-log of survival plot and Schoenfeld residuals^32^ were performed for proportional hazards assumption departure checking. To evaluate the survival of patients with different VA types, the proportional hazards regression model using two dummy variables for three types of VA was conducted to estimate the hazard ratio (HR) after adjustments for potential associated factors and the group using AVF/AVG only was defined as reference group. The univariate Cox proportional hazards regression model was used to assess the individual characteristics, baseline parameters, and VA effects on mortality. The parsimonious multivariable Cox proportional hazards regression model was developed to adjust potential factors by stepwise selection approach that included variables with significant criteria 0.1 for entry and 0.05 for removal. The reported p-values in our study were two-sided and required with significant level 0.05. All statistical analysis was carried out by SAS software Version 9.4.

## Supplementary information

Supplement Figure S1 and S2
