# Supplementary material for: The Impact of Vascular Access Types on Hemodialysis Patient Long-term Survival
Source: Sci Rep. 2019 Jul 24;9:10708. doi: 10.1038/s41598-019-47065-z (PMC6656721; doi:10.1038/s41598-019-47065-z)
Supplement: Supplementary file 1 — Supplement Figure S1 and S2 [file 41598_2019_47065_MOESM1_ESM.pdf]

The Impact of Vascular Access Types on Hemodialysis Patient Long-term Survival

Running: all-cause mortality and vascular access types

Li-Mei Yeh[1,2*], Sherry Yueh-Hsia Chiu[2,3*], Ping-Chin Lai[4,5]

1. Hemodialysis unit, Department of Nephrology, Chang Gung Memorial Hospital, Taipei, Taiwan
2. Department of Health Care Management and Healthy Aging Research Center, Chang Gung University, Taoyuan, Taiwan
3. Division of Hepatogastroenterology, Department of Internal Medicine, Kaohsiung Chang Gung Memorial Hospital, Kaohsiung, Taiwan
4. Department of Nephrology, Chang Gung Memorial Hospital, Taoyuan, Taiwan
5. The Kidney Institute and Division of Nephrology, China Medical University Hospital, Taichung, Taiwan

*: equally contributed

Corresponding authors: Dr. Ping-Chin Lai, The Kidney Institute and Division of Nephrology, China Medical University Hospital.

Address: No. 2, Yu-Der Road, North District, Taichung City, Taiwan 40447

Tel.: +886- 4-22052121ext 7387; E-mail: georgepclai@gmail.com

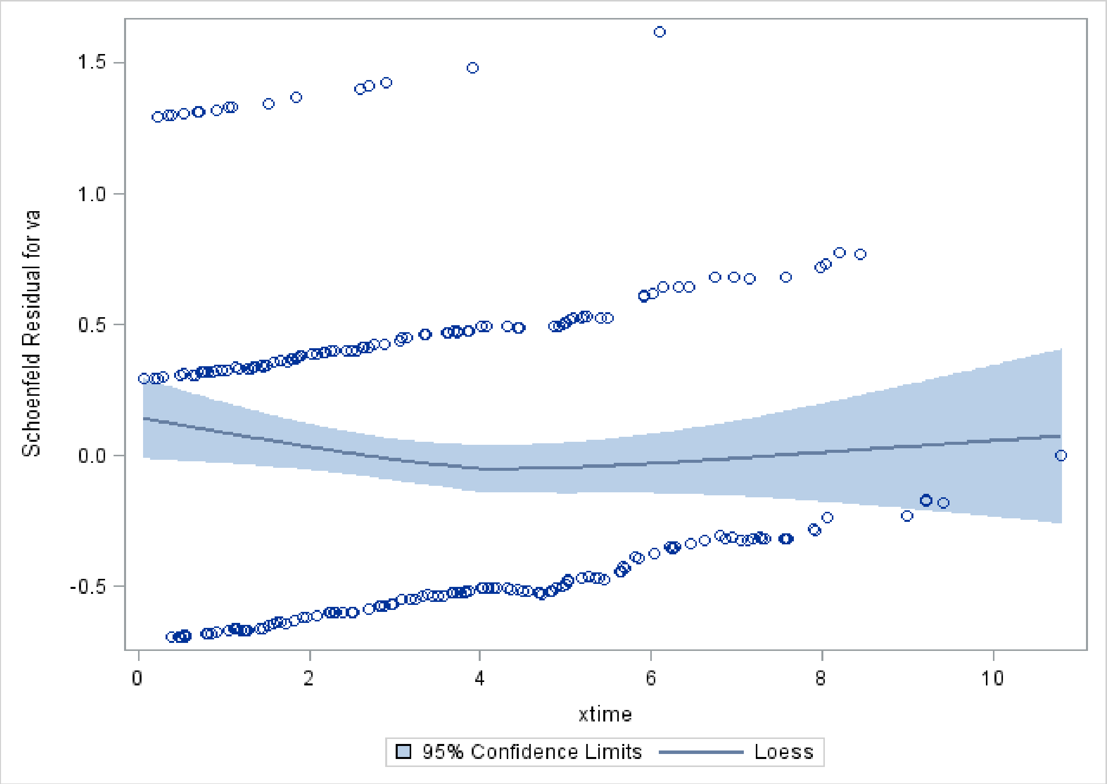

Figure S1. The result of proportional hazards assumption test based on Schoenfeld residuals

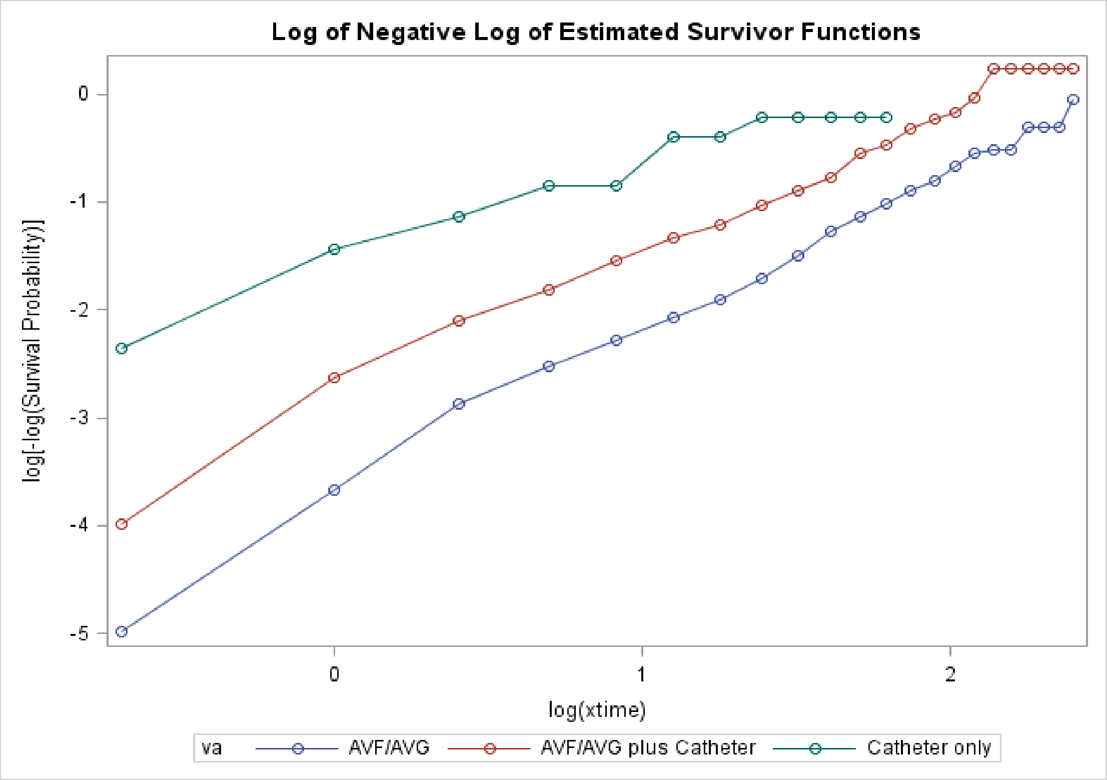

Figure S2. The result of proportional hazards assumption test based on log-negative-log of survival
